# Supplementary material for: Asynchronous magnetic resonance elastography: Shear wave speed reconstruction using noise correlation of incoherent waves
Source: Magn Reson Med. 2022 Oct 27;89(3):990–1001. doi: 10.1002/mrm.29502 (PMC9792433; doi:10.1002/mrm.29502)
Supplement: Supplementary file 1 — DATA S1 MATLAB code used to generate the numerical simulations of Figure 1 [file MRM-89-990-s002.zip › k-Wave/helpfiles/scanConversion.html]

scanConversion :: Functions (k-Wave)


# scanConversion

Convert scan-lines in polar coordinates to a B-mode ultrasound image.

## Syntax

```
b_mode = scanConversion(scan_lines, steering_angles, image_size, c0, dt)
b_mode = scanConversion(scan_lines, steering_angles, image_size, c0, dt, resolution)
```

## Description

`scanConversion` computes the remapping between a series of scan lines in polar coordinates (i.e., taken at different steering angles) to a B-mode ultrasound image in Cartesian coordinates suitable for display. The remapping is performed using bilinear interpolation via `interp2`.

## Inputs

|  |  |
| --- | --- |
| `scan_lines` | matrix of scan lines indexed as (angle, time) |
| `steering_angles` | array of scanning angles [degrees] |
| `image_size` | [x, y] size of the desired output image [m] |
| `c0` | sound speed in the medium [m/s] |
| `dt` | time step used in the acquisition of the scan lines [s] |
| `resolution` | optional input to set the resolution of the output images in pixels (default = [256, 256]) |

## Outputs

|  |  |
| --- | --- |
| `b_mode` | the converted B-mode ultrasound image |

## See Also

`interp2`
